# Transcriptome profiling of *Variovorax paradoxus* EPS under different growth conditions reveals regulatory and structural novelty in biofilm formation

**DOI:** 10.1099/acmi.0.000121

**Published:** 2020-04-03

**Authors:** Richard J. Fredendall, Jenny L. Stone, Michael J. Pehl, Paul M. Orwin

**Affiliations:** ^1^​ Biology Department, California State University San Bernardino, San Bernardino, CA, USA

**Keywords:** *Variovorax paradoxus*, Transcriptome analysis, Biofilm

## Abstract

We used transcriptome analysis by paired-end strand-specific RNA-seq to evaluate the specific changes in gene expression associated with the transition to static biofilm growth in the rhizosphere plant growth-promoting bacterium *Variovorax paradoxus* EPS. Triplicate biological samples of exponential growth, stationary phase and static biofilm samples were examined. DESeq2 and Rockhopper were used to identify robust and widespread shifts in gene expression specific to each growth phase. We identified 1711 protein-coding genes (28%) using DESeq2 that had altered expression greater than twofold specifically in biofilms compared to exponential growth. Fewer genes were specifically differentially expressed in stationary-phase culture (757, 12%). A small set of genes (103/6020) were differentially expressed in opposing fashions in biofilm and stationary phase, indicating potentially substantial shifts in phenotype. Gene-ontology analysis showed that the only class of genes specifically upregulated in biofilms was associated with nutrient transport, highlighting the importance of nutrient uptake in the biofilm. The biofilm-specific genes did not overlap substantially with the loci identified by mutagenesis studies, although some were present in both sets. The most highly upregulated biofilm-specific gene is predicted to be a part of the RNA degradosome, which indicates that RNA stability is used to regulate the biofilm phenotype. Two small putative proteins, Varpa_0407 and Varpa_3832, are highly expressed specifically in biofilms and are predicted to be secreted DNA-binding proteins, which may stabilize extracellular DNA as a component of the biofilm matrix. An flp/tad type-IV pilus locus (Varpa_5148–60) is strongly downregulated specifically in biofilms, in contrast with results from other systems for these pili. Mutagenesis confirms that this locus is important in surface motility rather than biofilm formation. These experimental results suggest that *V. paradoxus* EPS biofilms have substantial regulatory and structural novelty.

## Introduction

It is well established that many if not most bacteria spend a large fraction of their time in the biofilm state [1]. Many models of biofilm formation and growth have been evaluated, and while there are differences in the outcomes [2], one widely accepted static biofilm model is attachment to an abiotic surface while immersed in growth medium [3]. This model has been used to study biofilm formation in pathogens, plant-associated bacteria and industrial biofouling, and life attached to natural surfaces (for reviews see [1, 4]). Biofilm gene-expression patterns have been examined in many systems – a search of the sequence read archive (SRA) for the terms ‘transcriptome and biofilm’ yields 922 results. The rapid advancement of DNA sequencing and reduction in cost have made genomes and transcriptomes much more widely available beyond the traditional model micro-organisms [5]. Transcriptome analysis can lead to insights into an organism’s response to changing physiological conditions that evade traditional mutagenesis approaches [6]. The costs for these analyses have dropped precipitously, and the number of bacterial whole genomes available is very large (74 764 on 12 October 2019, https://img.jgi.doe.gov/cgi-bin/m/main.cgi?section=ImgStatsOverview), especially in the easily cultivatable groups. This makes it possible to use these approaches outside the common model organisms to study complex phenotypes such as biofilm formation. Shifts in gene expression have been specifically associated with the biofilm lifestyle, across many different bacteria, and in different biofilm contexts [3, 7–9]. A wide distribution of gene-expression patterns have been observed, and there is a great deal of variability that is both strain specific and driven by experimental setup.

*Variovorax paradoxus* is a soil-dwelling member of the beta-proteobacteria that is widely recognized as an important plant-growth-promoting bacterium [10, 11]. It has been frequently identified as a degrader of xenobiotics and source of important enzymes for biocatalysis [10], and is frequently identified in animal- and human-associated microbiomes, particularly as part of the human oral microbiota [12]. Several finished genomes of *V. paradoxus* strains are published [13, 14], and many more are available as permanent draft sequences (20 annotated genomes including four complete genomes at http://www.ncbi.nlm.nih.gov). In spite of the biotechnological and agricultural relevance of this genus, there has been very little work done on the physiology, genetics or development of *V. paradoxus* or its close relatives. Many studies on the plant-growth-promoting activities of *Variovorax* have been performed (for example see [15, 16]), but none of them have focused on the molecular nature of the plant–microbe interaction. Naturally occurring biofilms containing *V. paradoxus* have been studied *in situ,* and studies on biotechnologically relevant bioreactor cultures have been evaluated extensively [17–20]. Detailed studies on the genetics and biochemistry of *V. paradoxus* biofilms are unfortunately sorely lacking. Since like most micro-organisms *V. paradoxus* is commonly found growing in a biofilm, the paucity of these studies is an impediment to the effective exploitation of their metabolic capacity. Laboratory studies in *V. paradoxus* EPS have been performed to identify the conditions that are conducive to swarming motility and biofilm formation [21], and we have also used insertional mutagenesis to identify some genes involved in these complex phenotypes [22].

The shift to biofilm growth in *V. paradoxus* is accompanied by a large-scale change in transcript profile that differs greatly from the shift to stationary-phase growth. A total of 1711 transcripts were found to be uniquely and significantly altered in expression by more than twofold in the biofilm cultures, while 757 transcripts were similarly specific to stationary-phase growth. Using two different computational approaches, we identify structural and regulatory elements that are potentially critical to *V. paradoxus* biofilms, including a potential model of regulation based on RNA stability, and a DNA-binding protein that may stabilize and protect the biofilm matrix. Finally, we note an unusual pattern of expression in the putative flp/fap pilus locus, and using complementation analysis provide evidence that this locus is being utilized in motility rather than sessile attachment as described in other systems.

## Methods

### Culture conditions

All cultures were grown in 2.5 g l^−1^ yeast extract (50 % YE) for 24 h from isolated colonies picked from a low-passage plate (1–2 passages since −80 °C storage). All cultures were incubated at 30** **°C at all times, and liquid growth cultures were incubated with shaking at 200 r.p.m. for maximal oxygenation. For logarithmic growth, the culture was diluted 1 : 20 into 10 ml of 50 % YE, and monitored spectrophotometrically at OD595. Aliquots from three separate cultures were collected when the OD595 was approximately 0.5, and RNA was immediately extracted from ~10^9^ cells per sample. Identical cultures were grown for stationary-phase analysis, but RNA was collected when the OD595 was stable for successive readings spaced 30 m apart (approximately 18 h after dilution inoculation). For biofilm cultures, an identical dilution was grown for 24 h in 12-well Falcon plates (non-tissue culture treated) with 2 ml of liquid medium per well. The plates were incubated at an angle using a 10 ml serological pipet to create an air/liquid interface on the bottom of the well. After 24 h the medium was replaced with minimal disruption to the biofilm, and the culture was incubated for an additional 24 h under the same conditions. After this incubation the liquid medium was removed, the plate was washed with fresh medium, and the biofilm was recovered by scraping. One well in the plate was incubated with media only as an inoculum control. The 11 inoculated wells from each plate constituted a biological replicate.

### RNA isolation

Samples were pelleted by centrifugation at 10 000 ***g*** and the supernatant was discarded. The pellet was resuspended in 200 µl of 1 mg ml^−1^ lysozyme in 10 mM Tris 1 mM EDTA (TE, pH 8.0). Two volumes of RNAprotect (Qiagen) was added to each sample and the samples were incubated for 10 min at 25 °C. Some samples were preserved in this solution at −80 °C until further processing. The remaining steps were performed following the RNeasy (Qiagen) protocol. Successive addition of 700 µl of Buffer RLT and 500 µl of 100 % ethanol (RNA grade, Fisher) was followed by transfer of the mixture into an RNeasy Mini Spin column. After centrifugation the flow through was discarded. The column was washed with 350 µl of buffer RW1. To eliminate DNA from the sample, 100 µl of RQ-1 RNase-free DNase (Promega) was added to the column and incubated at 25 °C for 15 min. The column was then washed again with 350 µl of RW1 and the remaining steps of the protocol were followed.

### RNA purification and assessment

RNA was precipitated by adding 10 % (v/v) 3M sodium acetate, 5 µg glycogen and 3 volumes of 100 % ethanol, and incubating overnight at −20 °C. The RNA was pelleted by centrifugation in an Eppendorf 5810R centrifuge using a FA 45-30-11 rotor at 12 000 ***g*** for 30 min at 4 °C. The liquid was carefully removed by aspiration and the pellet resuspended in 1 ml of 70 % ethanol. After an additional identical centrifugation step, the pellet was dried and then resuspended in 24 µl of TE. The concentration and purity of the RNA samples were determined spectrophotometrically using a NanoDrop ND1000 spectrophotometer (Thermo Fisher). Further analysis for sample suitability was performed by Beijing Genomics Institute (Hong Kong).

### RNA-seq

The RNA was sequenced using a strand-specific paired-end protocol by BGI genomics (Hong Kong). Each sample was sequenced for 91 cycles in an Illumina HiSeq instrument with a total of 1.5×10^7^ reads per sample. Each of the three conditions (logarithmic, stationary, biofilm) was sampled in triplicate (biological replicates), derived from single colonies plated on YE agar directly from the original *V. paradoxus* EPS stock culture. The raw sequences were transmitted as fastq files for subsequent analysis. These files have been uploaded to NCBI and are available as gzip archives (BioProject PRJNA594416, BioSample SAMN13517278 , SRA accession #s SRR10613920-8).

### Expression analysis

Raw-sequence data were uploaded to the Galaxy main server (galaxy.psu.edu) and all of the bioinformatic tools described below were accessed at that site, unless otherwise noted. Uploaded sequences were trimmed based on quality using the Trimmomatic tool [23], and aligned to the *V. paradoxus* EPS genome using BWA [24]. Trimmed sequences were also aligned to the genome using the Rockhopper suite of RNA-seq tools for differential gene-expression analysis [25] on a local desktop computer. The BWA alignment was transformed into count data using the StringTie tool [26], and differential expression analysis was undertaken using the DESeq2 version 1.18.1 toolkit following the workflow outlined in Love *et al*. [27]. The DESeq2 program treated each individual gene as a separate transcript for the purposes of differential expression analysis. Venn diagrams were drawn in the Galaxy tool using the output of DESeq2 filtered on >2 × change in expression level, and tables of up and downregulated genes were generated using the Filter tool on Benjamini–Hochberg adjusted *P*-value *P*<0.05 and fold expression change >2. Transcript counts from Rockhopper were visualized (.wig files) in the Integrated Genome Viewer (IGV) available at (https://software.broadinstitute.org/software/igv/). Additional Rockhopper text file output on operon structure and *de novo* transcript structure were used for analysis of specific individual genes.

### Motility mutant screen and complementation

Transposon mutants were generated using Tn5 (TetR) as described previously [22]. Mutants with impaired motility were enriched in YE (5 g l^−1^) liquid culture by growth with shaking overnight followed by a 1 h settling period, followed by transfer of 1 : 100 of the culture volume into a fresh tube. This procedure was repeated five times, followed by plating on swarming medium [21]. Isolates that were identified as having decreased motility were subsequently tested for stability of the defect by repeated swarming assays. The interrupted gene was identified by the rescue cloning approach described previously [22]. PCR-amplified fragments from the *V. paradoxus* EPS genome corresponding to Varpa_5148 alone and the region including Varpa_5148–50 were cloned including regulatory sequence identified from transcriptome examination into pCR2.1 (KanR) (Invitrogen) and verified by Sanger sequencing. These elements were subcloned into pBBR1MCS-2 (KanR) [28] using *Bam*HI and *Hin*dIII sites incorporated into the primers. The complementation constructs and control plasmids were then introduced into *V. paradoxus* EPS by electroporation as described previously [22].

## Results and discussion

### Differential expression analysis using DESeq2

**Fig. 1.**
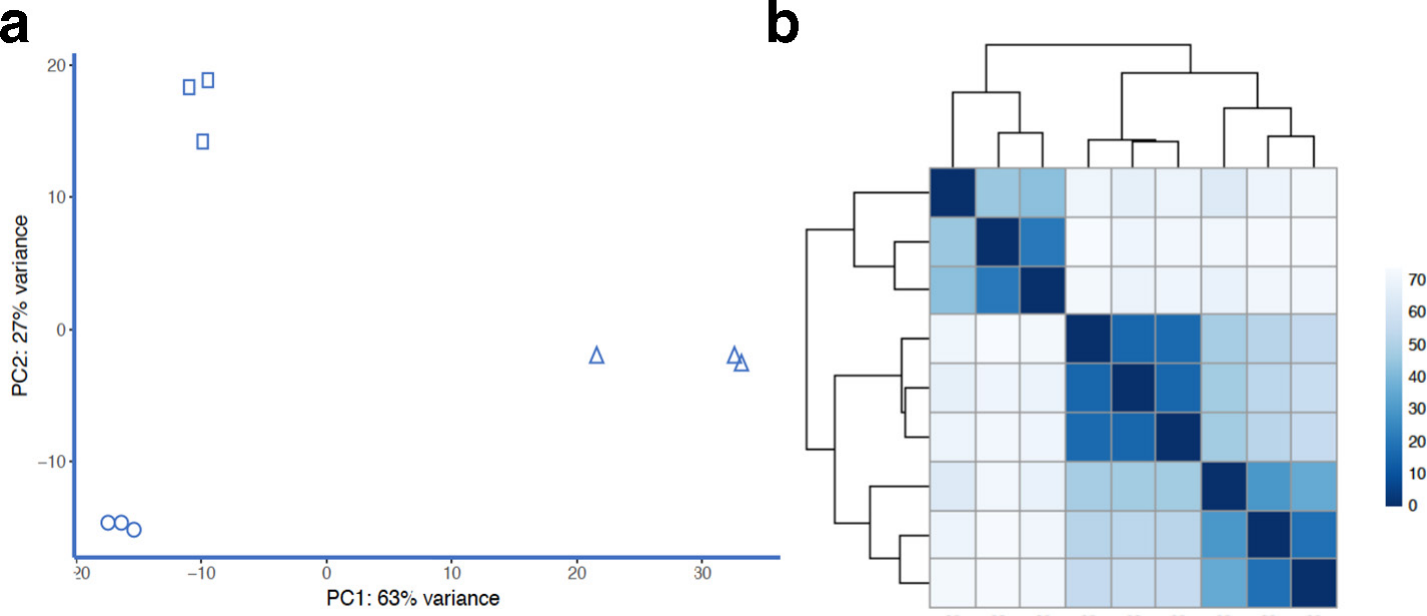
(a) PCA analysis of datasets using DESeq2 shows clustering of Log phase (circles), stationary phase (squares) and biofilm (triangles) samples. (b) Heatmap of Euclidian distance shows similar clustering of samples, indicating biological replicate consistency.

**Fig. 2.**
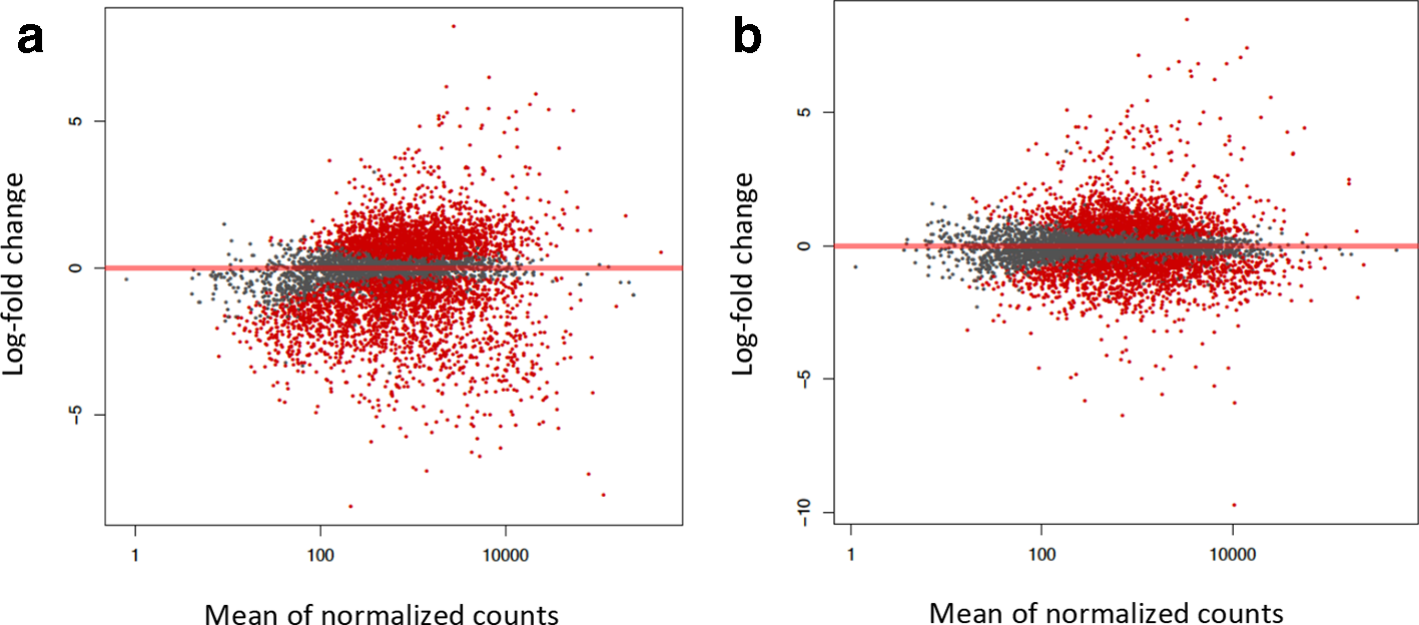
MA plots. (a) Exponential planktonic growth compared to biofilms. (b) Exponential planktonic growth compared to stationary phase. Red dots indicate adjusted *P*-value (*Q*-value) of <0.1.

**Fig. 3.**
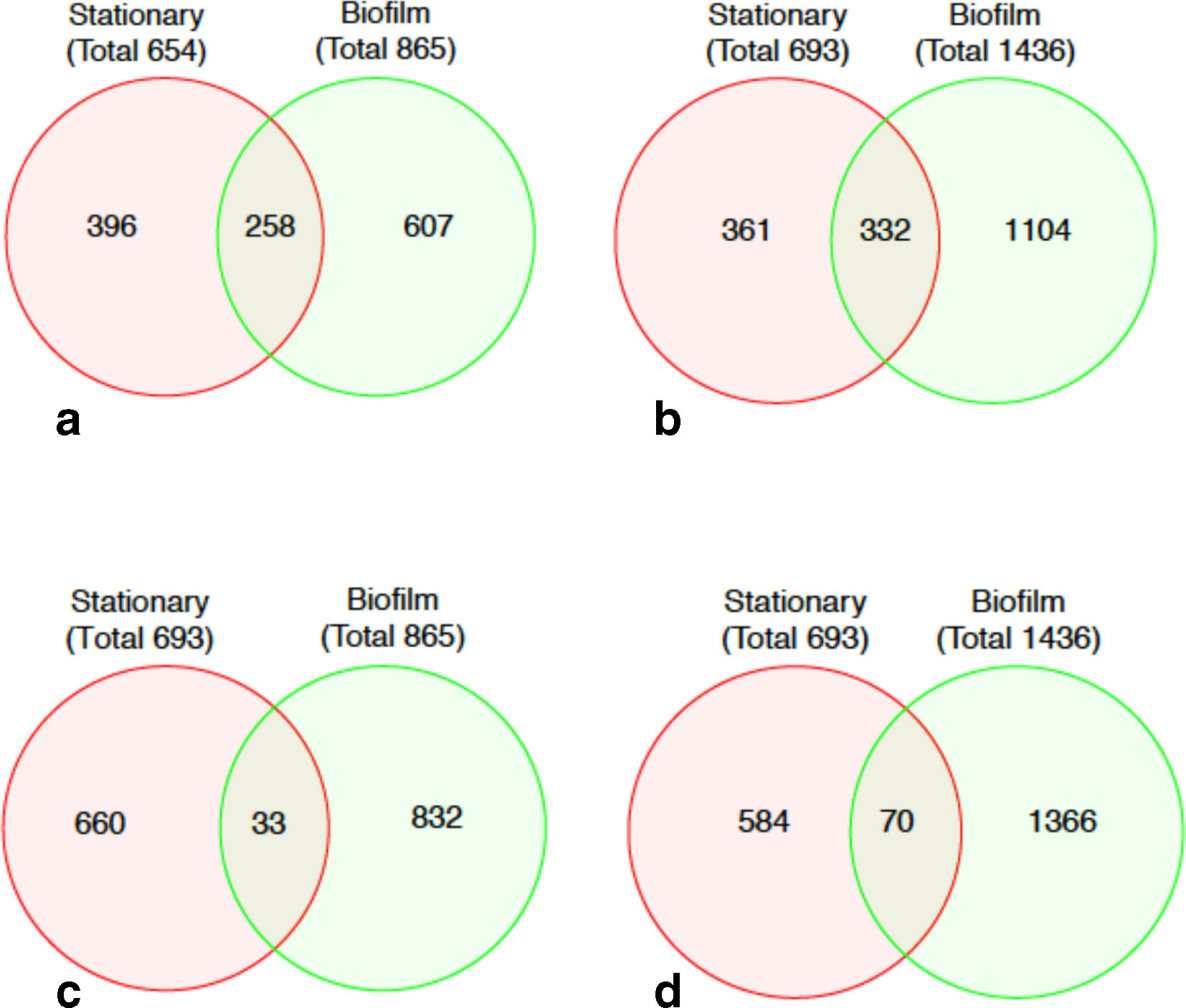
Venn diagrams of genes differentially expressed in stationary phase or biofilm compared to exponential growth. Output of the DESeq2 analysis pipeline were filtered using twofold expression as a cutoff. (a) Genes downregulated twofold or more. (b) Genes upregulated twofold or more. (c) Genes upregulated in stationary phase and down in biofilm. (d) Genes downregulated in stationary phase and upregulated in biofilm.

The DESeq2 suite of programs was used to analyse transcripts from all three growth conditions (exponential growth, stationary phase, biofilm) in a pairwise fashion [27]. All of the outputs of DESeq2 are included in Supplementary Data Sheet 1 (S1). The three sets of samples were evaluated for sample consistency among biological replicates using PCA analysis (Fig. 1a) and a heatmap of Euclidian distance (Fig. 1b). The three biological replicates from each sample are clustered together as expected, and exponential growth samples (‘log’) are less variable than either the stationary phase or biofilm samples. Using the Benjamini–Hochberg false discovery rate, the adjusted *P*-values were calculated for each pairwise comparison. The MA plots of comparisons between log phase and either stationary phase (Fig. 2a) or biofilm (Fig. 2b) show the distribution of differential expression. The top 25 up and downregulated loci unique to biofilm growth are listed in Tables 1 and 2, respectively. Loci identified by DESeq2 but not corresponding to an ORF were ignored for this analysis but included in the global expression analysis. After filtering for >2 × change in expression, 1104 loci were identified as having significantly increased expression uniquely during biofilm growth, while 607 had decreased expression using the same parameters (Fig. 3a, b). Interestingly, the unique proportion of the differentially regulated genes in each direction (1104/1436 v 607/865) was similar. An additional set of comparisons revealed a total of 103 genes where expression relative to exponential growth was regulated in opposite directions between biofilm and stationary phase (Fig. 3c, d). The lists of genes specifically differentially expressed in biofilm and stationary phase, and the list of genes with opposite regulation, are listed in Supplementary Data Sheet 1 along with Reads Per Kilobase of transcript, per Million mapped reads (RPKM) values and DESeq2-derived significance values.

**Table 1.** Top 25 uniquely upregulated loci in biofilm cultures

Locus	Fold increase	Gene annotation
**Varpa_1640**	232.40	DEAD/DEAH box helicase domain protein
**Varpa_1020**	106.32	glycosyl transferase family 2(EC:2.4.1.-)
**Varpa_2951**	78.22	hypothetical protein
**Varpa_4530**	74.94	hypothetical protein
**Varpa_2932**	64.28	oxidoreductase faD-binding domain protein(EC:1.18.1.3)
**Varpa_2061**	45.16	tonb-dependent siderophore receptor
**Varpa_3832**	44.56	snf2 superfamily protein
**Varpa_5795**	42.19	hypothetical protein
**Varpa_0407**	41.93	snf2 superfamily protein
**Varpa_4034**	40.44	heat shock protein hsp20
**Varpa_4531**	39.90	magnesium-translocating p-type atpase(EC:3.6.3.2)
**Varpa_5608**	37.32	clr6; predicted protein of clr family
**Varpa_5794**	35.40	hypothetical protein
**Varpa_5215**	35.03	hypothetical protein
**Varpa_5106**	33.98	trove domain-containing protein
**Varpa_0109**	32.86	benzoyL-CoA-dihydrodiol lyase(EC:4.2.1.17)
**Varpa_5796**	30.39	hypothetical protein
**Varpa_3781**	30.18	hypothetical protein
**Varpa_3060**	29.45	hypothetical protein
**Varpa_5951**	28.71	dihydrodipicolinate synthetase(EC:4.2.1.52)
**Varpa_0468**	28.14	hypothetical protein
**Varpa_1128**	27.39	cyanophycin synthetase(EC:6.-)
**Varpa_3061**	27.12	hypothetical protein
**Varpa_5797**	26.16	yiaab two helix domain-containing protein
**Varpa_2489**	24.51	phosphate ABC transporter, periplasmic phosphate-binding protein

**Table 2.** Top 25 uniquely downregulated loci in biofilm cultures

Locus	Fold repression	GENE annotation
**Varpa_4525**	18.64	extracellular solute-binding protein, family 7
**Varpa_1647**	12.43	indolepyruvate oxidoreductase subunit b(EC:1.2.7.8)
**Varpa_0430**	11.76	sigma 54 modulation protein/ribosomal protein s30ea
**Varpa_2331**	10.40	glycine cleavage system h protein
**Varpa_2332**	8.47	glycine cleavage system t protein(EC:2.1.2.10)
**Varpa_1832**	7.87	hypothetical protein
**Varpa_1660**	7.78	extracellular liganD-binding receptor
**Varpa_4079**	7.72	efflux transporter, rnd family, mfp subunit
**Varpa_5437**	7.37	hypothetical protein
**Varpa_4653**	7.27	aminotransferase class-iii(EC:4.1.1.64)
**Varpa_4408**	6.80	hypothetical protein
**Varpa_0874**	6.79	hypothetical protein
**Varpa_3019**	6.47	hypothetical protein
**Varpa_1470**	6.43	hypothetical protein
**Varpa_2596**	6.34	hypothetical protein
**Varpa_5615**	6.07	extracellular liganD-binding receptor
**Varpa_2330**	6.00	glycine dehydrogenase(EC:1.4.4.2)
**Varpa_5988**	5.88	hypothetical protein
**Varpa_4455**	5.85	hypothetical protein
**Varpa_4664**	5.75	two-component transcriptional regulator luxR family
**Varpa_2423**	5.74	hypothetical protein
**Varpa_1645**	5.71	transcriptional regulator marr family
**Varpa_4080**	5.70	outer membrane efflux protein
**Varpa_2366**	5.70	gcn5-related N-acetyltransferase
**Varpa_3579**	5.65	membrane alanyl aminopeptidase(EC:3.4.11.2)

### Transcriptome structure analysis with Rockhopper

Pairwise analysis of the transcriptome replicates was performed using Rockhopper [25] to compare the methods and evaluate the robustness of the datasets. The overall results of differential expression analysis were similar (not shown) with differences attributable to count normalization and annotation parameters. We limited our use of Rockhopper to in-depth analysis of individual loci and to identification of operon structure. This program will also identify small RNA, but since our initial data collection did not specifically isolate small RNA, data on differential small RNA expression is not presented here. Overall, 1262 multigene operons were identified by Rockhopper based on transcript data, along with 3021 gene pairs. The total number of protein-coding genes identified as differentially regulated in biofilms was 2145, while the number differentially regulated in stationary phase was 2508. This was a substantially different outcome than with DESeq2. When examining the text outputs of the comparison, it was observed that Rockhopper and DESeq2 both identify many potential small RNAs and other non-coding elements, which are not directly comparable. In all cases where an individual gene was evaluated for differential expression, the results were similar, and quantitative differences in fold-induction or repression are likely due to different normalization or *de novo* transcript identification algorithms. All of these results are included in Supplementary Data Sheet 2 (S2).

### Loci previously evaluated for biofilm transcription

Our previous work identified a number of loci that when mutated resulted in altered biofilm and/or swarming phenotypes [22]. We found that only 8/30 loci identified previously by transposon insertion [22] were differentially regulated at the level of transcription, and all of those loci were downregulated in biofilms. In that work several loci were identified by multiple insertions that were all associated with the phenotypic alteration, namely multiple insertions into Varpa_5900 and Varpa_4680, encoding the PilY1 tip adhesin and a glycosyl transferase, respectively. In both of these cases the RNA-seq analysis confirmed the data collected previously by quantitative Reverse Transcription Polymerase Chain Reaction (qRT-PCR) [22], confirming the validity of the assay conditions independently. Interestingly, there are 3 PilY1 loci in *V. paradoxus* EPS (5900, 3518, 4912), but only Varpa_5900 is expressed to a significant degree under any of the conditions tested.

### Gene ontology enrichment

**Fig. 4.**
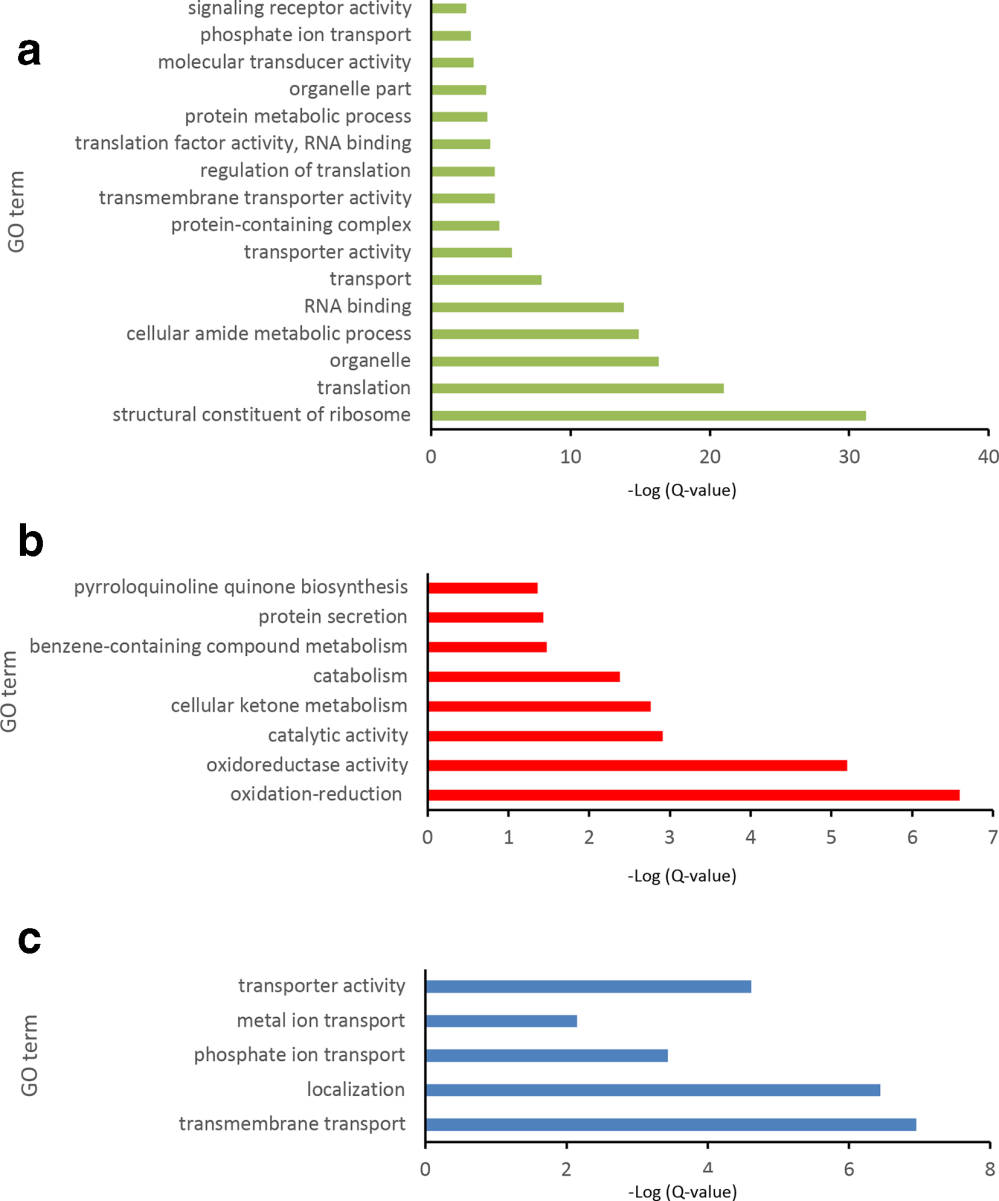
Analysis of biofilm upregulated gene classes (a) and biofilm downregulated gene classes (b) compared to whole genome. Panel (c) shows the GO enrichment of genes only upregulated in biofilm and not in stationary-phase cultures. No GO terms were found to be enriched in genes downregulated only in biofilms.

Gene-ontology analysis of the upregulated and downregulated loci is presented in Fig. 4. Panels (a) and (b) show diverse sets of gene categories that are overrepresented among the genes with differential expression in biofilms, while (c) identifies a much narrower set of genes overrepresented in the set of genes upregulated specifically in biofilms. No gene categories were overrepresented among the loci specifically downregulated in biofilms. The overrepresented categories in Fig. 4c are indicative of the increased need for transport of essential micronutrients such as metal co-factors and nutrients that tend to limit replication and viability such as inorganic phosphorus. This latter gene family may also be connected to the use of eDNA as a nutrient storage mechanism as well as a structural component of the biofilm matrix [29]. These GO category patterns are consistent with a biofilm structure with increased nutrient transport to maintain viability throughout the structure and to distribute resources throughout the biofilm.

### Secreted snf2 family proteins

**Fig. 5.**
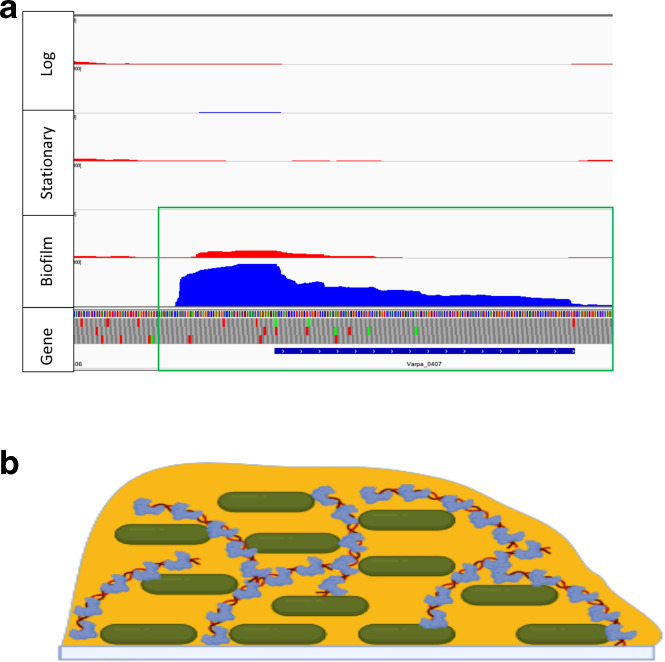
(a) Varpa_0407 transcriptome data showing leader region, antisense expression only in biofilm growth (green box). Blue and red colouring signals transcripts in the sense and antisense directions, respectively. (b) model of biofilm eDNA protection by Snf2 superfamily secreted proteins.

A pair of small proteins were specifically highly upregulated in biofilm growth (Table 1, red), which have high homology to one another (Varpa_0407 and Varpa_3832, 83 % identity between amino acid sequences). Highly similar proteins are found in some other *V. paradoxus* genomes examined, but no close orthologues were found outside the genus using blastp [30]. Both genes are highly expressed specifically in the biofilm state, and both have an extensive 5′ untranslated region (Fig. 5a, only Varpa_0407 shown). They are annotated as members of the Snf2 superfamily of DNA-binding proteins [31, 32], but were much smaller than all previously described proteins in this superfamily. These Snf2 proteins in *V. paradoxus* are also annotated as having a 24 amino acid leader peptide cleaved in each case to generate a mature 102 amino acid protein. The most common association of Snf2 proteins with function is with helicase activity and chromatin remodelling [32], neither of which is compatible with a bacterial secreted protein. Our conjecture based on this information is that these small proteins are non-specific DNA-binding proteins that are secreted to stabilize the biofilm structure, which likely contains extracellular DNA (eDNA) as a structural component, as is common in many bacterial species [29]. The presence of these proteins may stabilize the biofilm matrix and protect against extracellular DNAse activity (Fig. 5b). It also may be that they were not uncovered by mutational analysis because of their likely functional redundancy.

### RNA turnover role in biofilm regulation

**Fig. 6.**
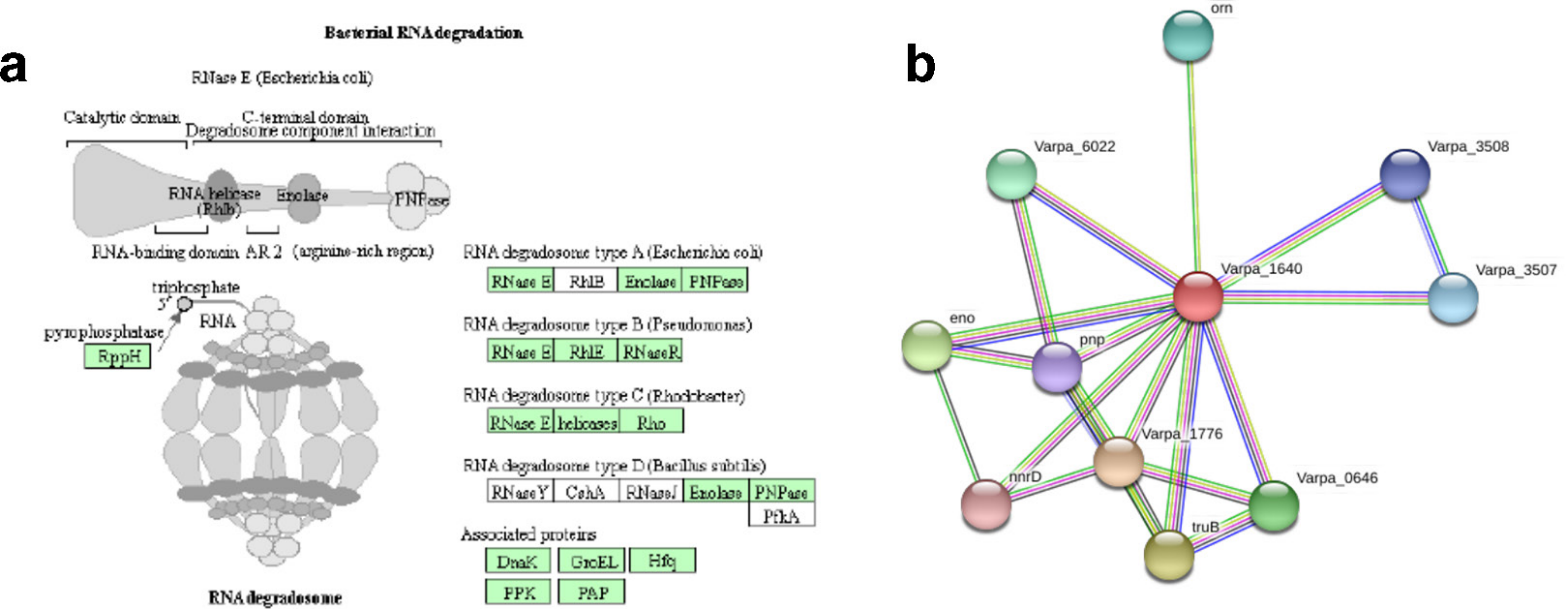
(a) Proposed role of Varpa_1640 in RNA turnover. Although it is not annotated as such, Varpa_1640 could fit functionally into the type A–C degradosomes in the place of RhlB, RhlE or unspecified helicases (b) STRING protein interaction network for Varpa_1640 showing associations with components of RNA degradosome.

The most specifically highly upregulated gene in our analysis was Varpa_1640 (Table 1, green), which was not identified in our previous biofilm mutant screen. This protein is predicted to be a DEAD-box RNA helicase, a widespread family of proteins recently shown to play a role in biofilm formation [33]. We hypothesize that this protein is active in RNA turnover in *V. paradoxus* EPS based on the KEGG predicted RNA degradation pathway [34] (Fig. 6a). The type A–C RNA degradosomes (representing machinery identified in *E. coli, Pseudomonas* and *Rhodobacter *, respectively) are overlapping but well represented in the *V. paradoxus* EPS genome, consisting of polynucleotide phosphorylase (PNPase, Varpa_4029), enolase (Varpa_2167), RNaseE/R (Varpa_1519, Varpa_3333) and helicases (DEAD/DEAH box, Varpa_4256, Varpa_0178, Varpa_2773), along with the RNA 5′ pyrophosphohydrolase RppH (Varpa_4825) and the Rho transcription termination factor (Varpa_2500). Varpa_1640 was not identified in the KEGG orthology as part of this machinery, nor was the RhlB protein present in the KEGG assignments (Fig. 6a) but the STRING network map [35] for Varpa_1640 connects this locus to the RNA degradosome (Fig. 6b). The other DEAD/DEAH box helicase (Varpa_4256) is upregulated 15× specifically in biofilms, while none of the other proteins involved in RNA degradation are specifically upregulated in this growth phenotype. The PNPase, enolase and RNAses are all highly expressed in biofilm (Supplementary Data S1 and S2), leading to the hypothesis that differential expression of Varpa_1640 is a mechanism for regulating RNA stability and drastically altering the gene expression profile. A relationship between a DEAD-box helicase and biofilm growth was recently described in the plant pathogen *Xanthomonas citri* [33], based on mutations in the HrpB helicase resulting in reduced expression of type-IV pili. Intriguingly, our prior work indicated that type-IV pili are critical for the switch between biofilm and swarming phenotypes in *V. paradoxus* EPS [22], suggesting a possible link between RNA stability, pilus formation and attachment phenotypes.

### Regulation of pilus formation and the *tad* locus

**Fig. 7.**
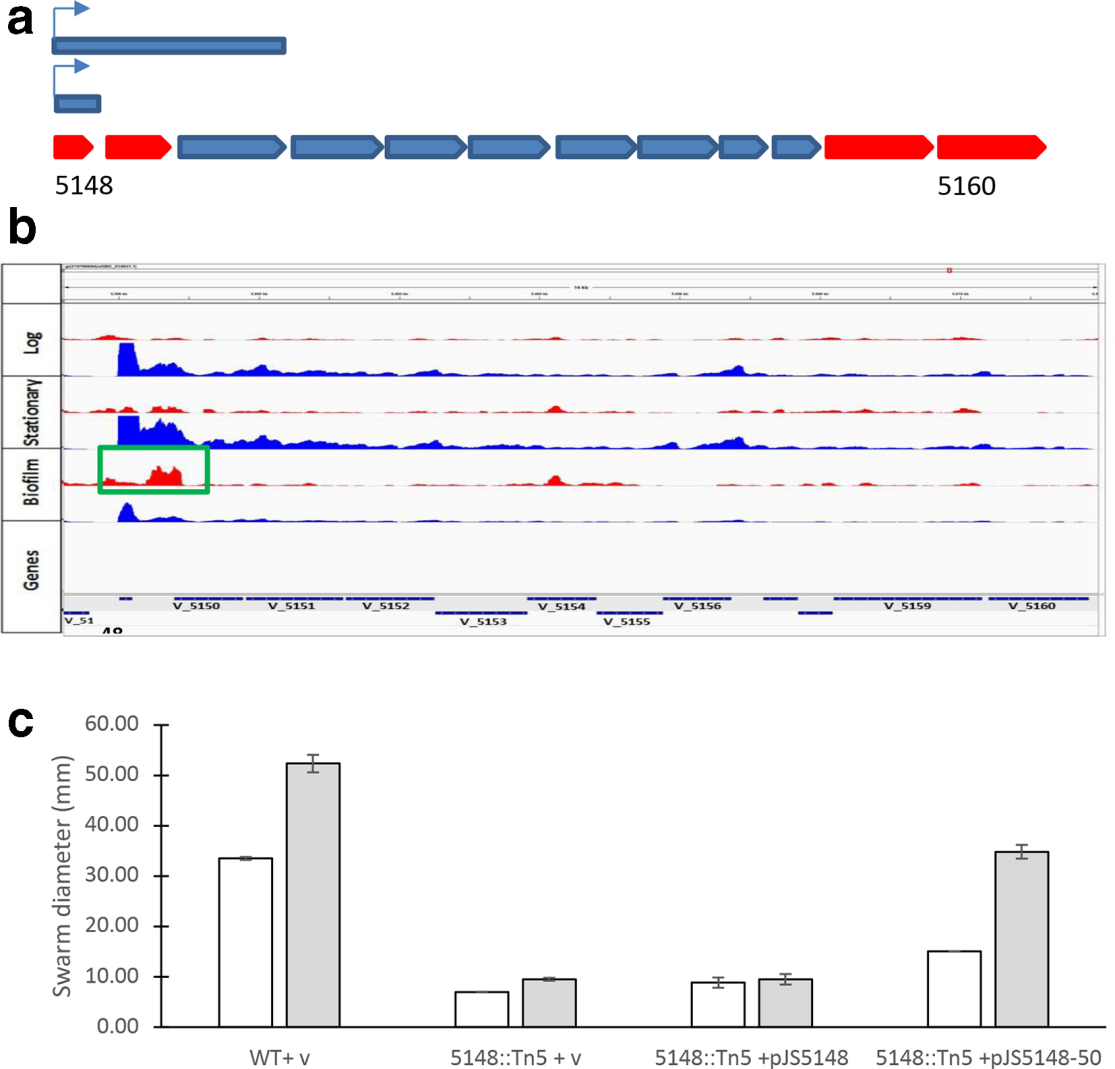
Putative tight adhesion pilus locus map and complementation. (a) Locus map and complementation constructs (blue bars). Red ORFs indicate sites where transposon insertions resulted in reduced motility. (b) Representative transcript profiles at the putative tad locus in all three tested phases of growth. A potential antisense transcript (green box) is associated specifically with biofilm growth. Blue and red colouring signals transcripts in the sense and antisense directions, respectively. (c) Complementation analysis suggests that the putative tad pilus is required for swarming motility.

The *V. paradoxus* EPS genome encodes multiple pili, including a type-IV pilus with multiple PilY1 proteins and a putative *tad*/*flp* pilus locus (Varpa 5148–5160) similar to the *tad* locus characterized in the bacterium *Aggregatibacter actinomycetemcomitans* [36]. Diverse roles in surface attachment and motility have been identified for pili in different bacterial systems especially type-IV pili (T4P) [37], making inference about the roles of these appendages difficult. The tad/flp pilin is classified as a T4Pb-class pilin, and is broadly distributed in both Gram-negative and Gram-positive bacteria [38]. This pilus has been identified as the appendage responsible for tight adhesion, and also as the bundle-forming pilus, and has been implicated in adherence to surfaces and virulence in several different bacteria [39–41]. In *P. aeruginosa *, pilus gene-expression changes in different strains and biofilm conditions can vary widely and be difficult to interpret [2, 42]. Inspection of the set of *V. paradoxus* EPS genes with altered expression for pilus-related loci revealed that all of the identified pilus genes were downregulated in biofilm with the exception of Varpa_3516 and Varpa_3520. However, the expression levels at these loci (which are predicted to be cotranscribed – see Supplementary Data. S2) are quite minimal, so it is not clear that this signal is meaningful. The *tad* locus genes are predicted to be expressed in three distinct transcripts (Supplementary Data S2), with only the pilus gene and the prepilin peptidase having a very large fold decrease in expression in biofilms (Supplementary Data S1). The putative prepilin peptidase (Varpa_5149) was annotated as a pseudogene initially but in more recent annotations has been identified as an ORF (not shown, https://www.ncbi.nlm.nih.gov/nuccore/NC_014931.1?report=graph). The putative operons for the rest of the tight adhesion pilus are also downregulated, but to a much lower degree than the pilin and peptidase (Fig. 7b). The presence of a strong antisense RNA signal in this operon (Fig. 7b, green box) suggests potential regulation by RNA stability, as this rise in potential double-stranded RNA formation corresponds with the lower transcript levels specifically in biofilm growth. An independent experiment attempting to enrich for motility mutants identified transposon insertions within the *tad* locus leading to deficiencies in swarming motility (Fig. 7a). Using a Tn5 insertion disrupting the Varpa_5148 pilin gene, we showed using complementation *in trans* with different constructs (Fig. 7a) that both the pilin and the prepilin peptidase (Varpa_5149) are necessary to restore motility in this background (Fig. 7c). Varpa_5150, which is predicted to encode a pilus assembly protein, was also present in the successful motility complementation construct. The construct was designed to ensure that the full prepilin peptidase gene would be included, and since the Varpa_5149 locus was annotated at the time as a pseudogene, it was unclear where the stop codon would be. In addition, the full length of the 5148–49 transcript was not known. The Varpa_5150 gene is not predicted based on transcriptome analysis to be part of the same transcript as the pilin or the prepilin peptidase and is predicted to encode a CpaB-type pilus assembly protein. It remains formally possible that the Varpa_5150 gene is the critical element for restoring surface motility. Further genetic analysis is required to make this determination. No change in biofilm phenotype was associated with this mutation, and no difference was seen between mutant, wild-type or complementation constructs in terms of biofilm formation. Our previous report on transposon mutagenesis altering swarming motility and biofilm formation in this system identified multiple loci that had impacts on both phenotypes, as well as some that had only impact on one [22]. However, that screen was based on an initial agar plate phenotype, and thus likely to overlook some mutants. This data supports experimentally the operon structure suggested by transcriptome analysis and is evidence that this pilus is directly involved in swarming motility, which has not previously been shown in any system. Previously, only *Pseudomonas aeruginosa* had been shown to contain both type-IVa and IVb pili [41], and this is the first time to our knowledge that the tad/flp pilus has been associated directly with motility.

## Conclusions

We show here that the rhizosphere isolate *V. paradoxus* EPS has a substantial shift in gene expression when it grows in a biofilm, with about 28 % of its genome specifically altered in expression in a static biofilm model. Analysis of the expression pattern has led to a potential new hypothesis about the role of RNA stability in this phenotype, and the specific role of the Varpa_1640 DEAD-box helicase in this mechanism. A previously unrecognized type of small secreted DNA-binding protein was identified and is proposed to have an important and specific role in biofilm growth. In contrast with previous work in other systems, the expression profile, presence of a cis-antisense RNA and mutational analysis suggest that the *flp/tad* locus is suppressed in biofilms and expressed during motile growth. Mutational analysis of biofilm formation yields sharply contrasting results compared to transcriptomic analysis, suggesting both methods are necessary for a complete picture of this complex phenotype.

## Supplementary Data

Supplementary material 1

Supplementary material 2
